# Mediator Complex Subunit MED1 Protein Expression Is Decreased during Bladder Cancer Progression

**DOI:** 10.3389/fmed.2017.00030

**Published:** 2017-03-17

**Authors:** Niklas Klümper, Isabella Syring, Wenzel Vogel, Doris Schmidt, Stefan C. Müller, Jörg Ellinger, David Adler, Johannes Brägelmann, Sven Perner

**Affiliations:** ^1^Pathology of the University Medical Center Schleswig-Holstein, Campus Luebeck and the Research Center Borstel, Leibniz Center for Medicine and Biosciences, Luebeck, Germany; ^2^Clinic for Urology and Pediatric Urology, University Hospital of Bonn, Bonn, Germany; ^3^Department of Hematology, Oncology and Rheumatology, University Hospital of Bonn, Bonn, Germany

**Keywords:** mediator complex, mediator, bladder cancer, MED1, immunohistochemistry

## Abstract

**Introduction:**

Bladder cancer (BCa) is among the most frequent cancer entities and relevantly contributes to cancer-associated deaths worldwide. The multi-protein Mediator complex is a central regulator of the transcriptional machinery of protein-coding genes and has been described to be altered in several malignancies. MED1, a subunit of the tail module, was described to negatively modulate expression of metastasis-related genes and to be downregulated in melanoma and lung cancer. In contrast, MED1 hyperactivity was described in breast and prostate cancer, likely due its function as a hub for nuclear hormone receptors. So far, only little is known about the function of the Mediator complex in BCa. The aim of this study was therefore to investigate the role of MED1 in BCa as a prognostic biomarker and a biomarker of disease progression.

**Methods:**

The protein expression of MED1 was assessed by immunohistochemistry (IHC) on tissue microarrays from 224 patients: benign urothelium *n* = 31, non-muscle invasive BCa (pTis, pT1) *n* = 72, and muscle invasive BCa (pT2–T4) *n* = 121. Comprehensive clinicopathological information including follow-up were available. Quantification of MED1 protein expression was evaluated by the semiquantitative image analysis program Definiens.

**Results:**

MED1 expression significantly decreased during BCa progression from benign urothelium to advanced BCa. Muscle invasion, the crucial step in BCa progression, was associated with low MED1 protein expression. Accordingly, decreased MED1 expression was found in primary BCa samples with positive lymphonodal status and distant metastases. Furthermore, cancer-specific survival was significantly worse in the group of low MED1 expression.

**Conclusion:**

Our findings show that the downregulation of MED1 is associated with muscle invasion, metastatic spread, and shorter overall survival in BCa.

## Introduction

Bladder cancer (BCa) is occurring frequently with about 180,000 new cases and 40,000 deaths per year in the European Union (1). It is the fifth most common entity in men in the Western population and the most frequent of the urinary tract (1, 2). To date, BCa contributes strongly to cancer-associated deaths worldwide, whereby the crucial step in BCa progression is the local invasion of the M. detrusor vesicae and distant metastatic spread (1, 2). Due to unspecific clinical symptoms, diagnosis of BCa frequently occurs at the muscle-invasive stage, which is often accompanied by distant metastases leading to unfavorable outcome. To improve the therapeutic management for patients suffering with BCa, a deeper understanding of the molecular biology of this cancer entity is necessary. Therefore, especially, the mechanisms of enhanced motility leading to muscle invasion and metastatic spread are of great interest to achieve better outcomes in the future.

Tight regulation of gene expression is important for physiological tissue integrity and cellular homeostasis. In cancer, this regulation is frequently altered. Tissue-specific expression of protein-coding genes by RNA polymerase II (Pol II) is therefore strictly controlled (3). The multi-protein Mediator complex (MED) is a main coactivator of transcription and globally regulates the Pol II (4). By building the bridge between transcriptional factors and the RNA Pol II, it is a central binding element integrating manifold transcriptional information (4, 5). In humans, the Mediator complex is a protein assembly of 33 subunits and largely conformationally dynamic through diverse subunit compositions (3–5). The Mediator complex consists of the four modules “head,” “middle,” “tail,” and “kinase,” and each of the MED subunits belongs to one of those modules. Deregulation of separate subunits has been recently linked to several malignancies (6, 7). There is great evidence for the kinase module CDK8 to function as an oncogene in colorectal carcinoma and its paralog CDK19 to be overexpressed during prostate cancer progression (8, 9). In melanoma, downregulation of the tail module MED1, also known as TRAP220, triggers a strong tumorigenic phenotype (10) and is associated with worse outcome in lung adenocarcinoma (11). Interestingly, loss of MED1 promotes the appearance of metastases of non-small-cell lung cancer cells by modulating metastasis-related genes (12) and was associated with the downregulation of tumor suppressor gene *dapk1* (13). Moreover, MED1 serves as a hub for nuclear receptors such as the estrogen (ER) or androgen receptor and has been linked to altered hormone receptor signaling in breast and prostate cancer (14–17). Nevertheless, only little is known about the role of the Mediator complex in BCa. Since MED1 has been described to be associated with metastatic spread repeatedly (10–13), this subunit is of high interest in this tumor entity, in which metastases and local invasion are crucial for patient outcome. We therefore investigated alterations of MED1 levels in BCa progression by immunohistochemical staining (IHC) in a large patient cohort with extensive clinical annotation including survival data.

## Materials and Methods

### Immunohistochemistry (IHC)

Ethical approval for using human material in the present study was obtained from the Internal Review Board of the University Hospital of Bonn (IRB# 036/08 and #093/12). The study participants were anonymized before their specimens were included to the study cohort.

Protein expression in paraffin-embedded BCa tissue was assessed on tissue microarrays (TMAs) from patient samples provided by the University Hospital of Bonn including benign bladder urothelium and BCa with different stages of disease [Table 1, benign urothelium *n* = 31, non-muscle invasive BCa (NMIBC; pTis, pT1) *n* = 72, and muscle invasive BCa (MIBC; pT2–T4) *n* = 121, in total *n* = 224]. Immunohistochemical staining was conducted on TMAs using the Ventana Benchmark automated staining system (Ventana Medical System, Tuscon, AZ, USA) as described previously (7, 9, 18, 19). In brief, slides were incubated at room temperature with the primary antibody: anti-TRAP220/MED1 rabbit polyclonal (dilution 1:500, ab64965, lot number: GR54026, Abcam). The testing of the antibody was conducted on breast carcinoma tissue as the positive control regarding to the manufacturer. Detection of the primary antibody was done with the ultraView Universal DAB detection kit (Ventana Medical System, Tuscon, AZ, USA). Finally, slides were counterstained with hematoxylin and bluing reagent, dehydrated, and mounted. IHC staining quality was validated independently by two experienced observers. Tumor samples with a lack of tissue on the TMAs were excluded.

**Table 1:** **Sample size (frequency) and clinicopathological data of the bladder cancer (BCa) cohort**.

	BCa Σ = 193	Benign Σ = 31
**Sex**		
Female	43 (22.3)	11 (35.5)
Male	150 (77.7)	20 (64.5)
**Age**		
Mean	67	65.6
Median	69	65
Range	36–94	43–81
**TNM**		
Tis	30 (15.4)	/
T1	42 (21.8)	/
T2	42 (21.8)	/
T3	42 (21.8)	/
T4	37 (19.2)	/
N1	22 (11.4)	/
M1	4 (2.1)	/
Muscle invasion	121 (62.7)	/
Cancer-associated death	46 (23.8)	/
Relapse	34 (19.7)	/

### Quantification of Protein Expression

MED1 nuclear expression was assessed digitally using the semiquantitative image analysis software Definiens Tissue Studio 2.1 (Definiens Inc., Munich, Germany) as described previously (7, 9, 14). Slides were scanned (Panoramic Desk, 3DHistech, Budapest, Hungary), and the tumor tissue in each sample was marked manually to exclude normal and stromal areas. For the benign samples tumor-free urothelium was also marked manually to differentiate between benign transitional epithelium and stroma. These user-specified regions of interest were analyzed by the Definiens software, and the average nuclear staining intensity (corresponding to the mean brown chromogen intensity) was quantified as a continuous value (arbitrary units) with higher values indicating stronger staining.

### Clinical Data and Statistics

Associations to available clinicopathological parameters were performed based on the MED1 expression intensities. After dividing the BCa patients by the mean MED1 expression for all tumor samples into two groups, survival analyses were evaluated by Kaplan–Meier estimator and log-rank test. Statistical evaluation was performed using Student’s *t*-test by SPSS and Microsoft Excel.

## Results

### Expression of MED1 in Tumor-Free Urothelium and BCa Tissue

**Figure 1:**
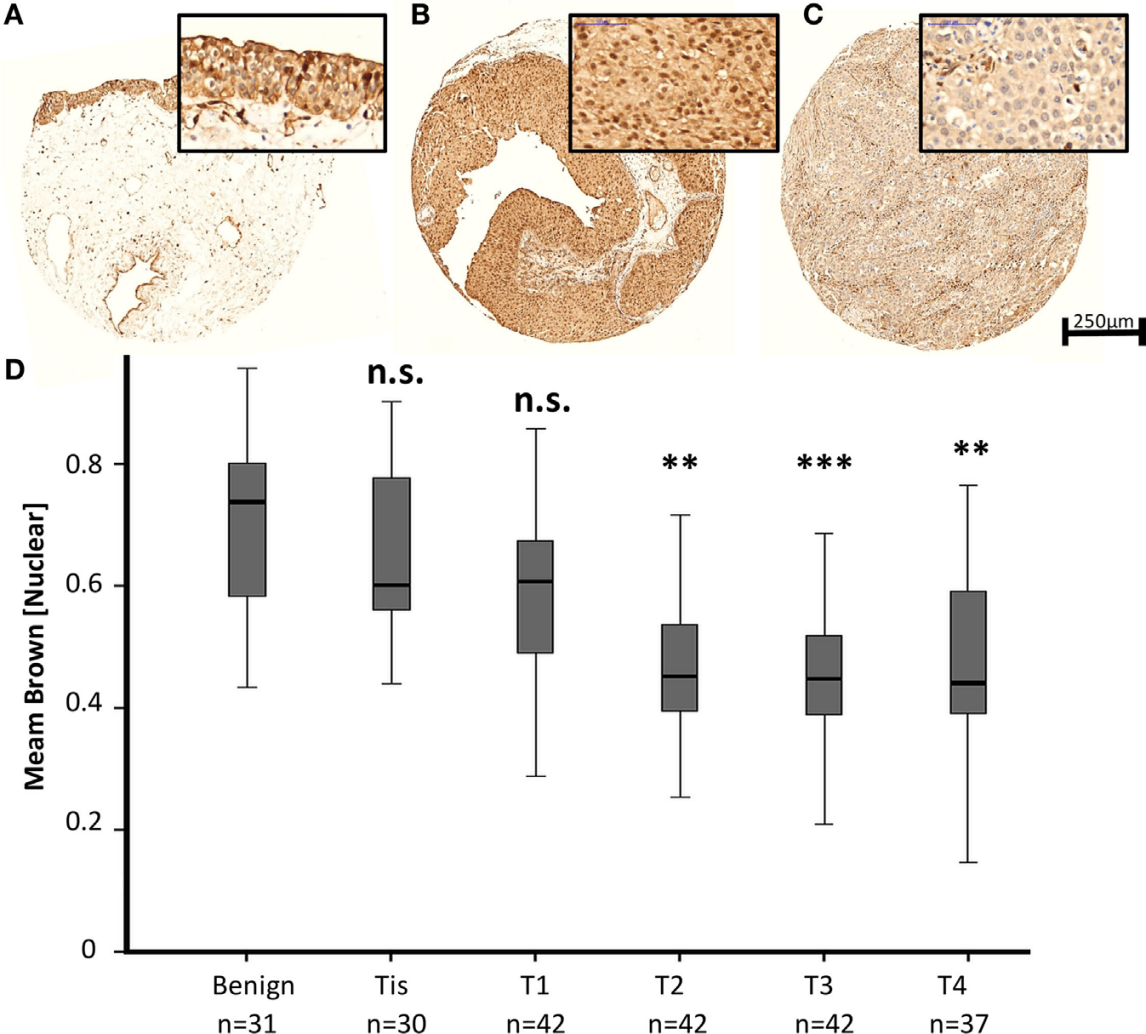
**Representative images of the MED1 immunohistochemical staining of benign urothelium (A), non-muscle invasive bladder cancer (BCa) [(B), pT1], and muscle-invasive BCa [(C), pT3], 5× and 40× objective magnification**. **(D)** Average MED1 staining intensity (mean brown chromogen intensity) of the BCa cohort across increasing tumor stages.

**Figure 2:**
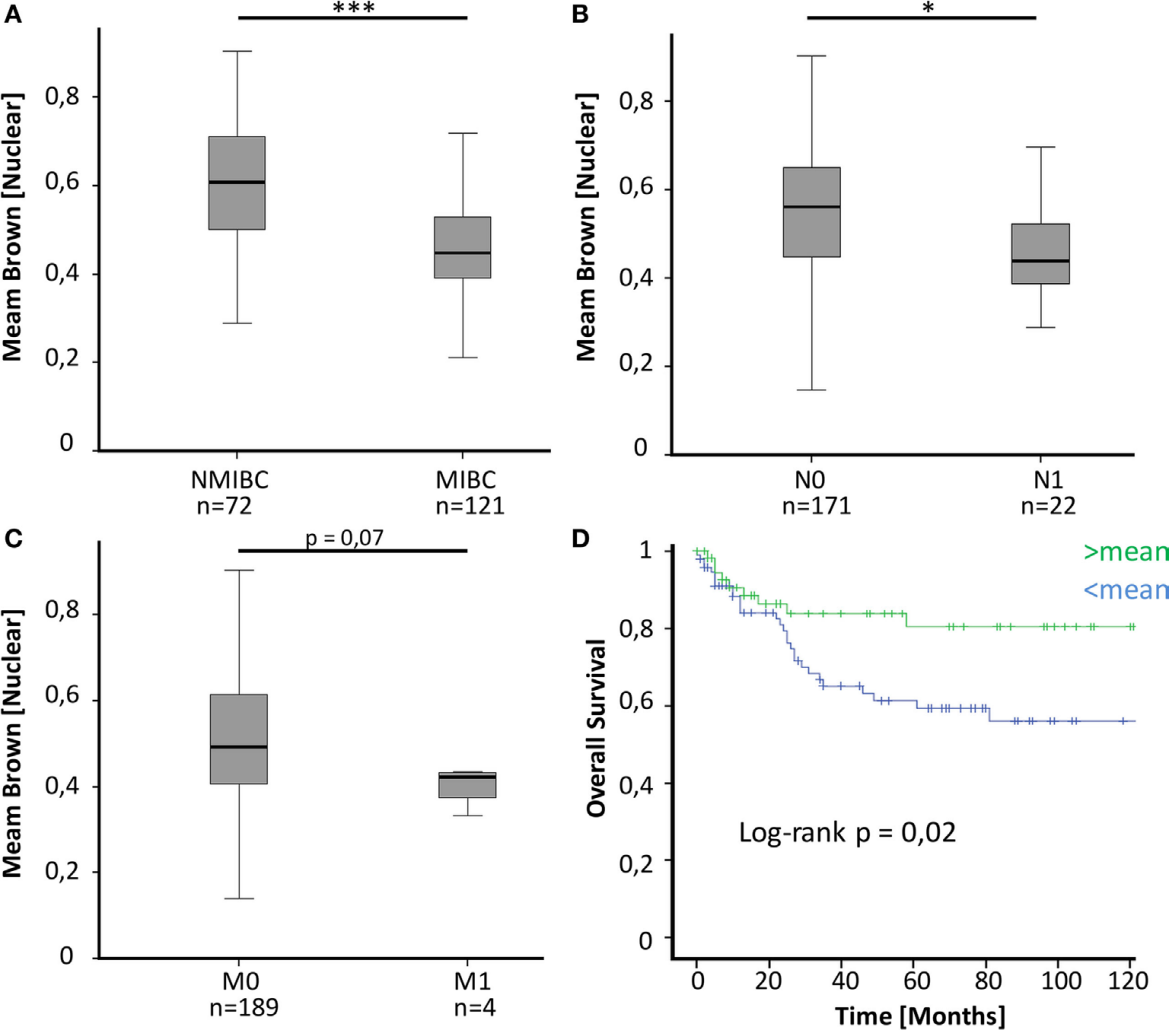
**(A)** MED1 expression is significantly lower in muscle-invasive bladder cancer (MIBC) compared to non-invasive pTis and T1 [non-muscle invasive bladder cancer (NMIBC)]. **(B,C)** Decreased MED1 expression is associated with positive lymphonodal status and distant metastases. N0/M0 = no lymphonodal or distant metastases, N1/M1 = positive lymphonodal status or distant metastases **(D)** The overall survival of BCa patients with low MED1 protein expression is worse compared to patients with high MED1 protein expression (low = <mean, high = >mean).

To evaluate a potential implication of MED1 protein expression during BCa development and progression, a large TMA including 224 BCa patient samples (Table 1) of all tumor stages of disease and benign urothelium was analyzed by immunohistochemistry (IHC) followed by computer-based semiquantitative image analysis. Mainly, MED1 protein expression showed a predominant nuclear localization, as the Mediator complex interacts in the nucleus with the transcriptional machinery. Additionally, MED1 was localized in the cytoplasm, where the MED1 protein is synthesized (Figures 1A–C). In tumor-free urothelium, the expression of MED1 was present in both cellular compartments, nucleus and cytoplasm (Figure 1A). Further, in NMIBC (NMIBC = pTis and pT1), the expression intensity of MED1 was similar compared to the benign transitional epithelium (Figure 1B, *p* = 0.99), whereas in muscle-invasive tumor stages (MIBC = T2–T4), the nuclear and cytoplasmic MED1 expression was significantly decreased compared to benign bladder tissue (Figure 1C, *p* = 5E−05). In total, the MED1 expression consistently decreased from benign urothelium and preinvasive carcinoma *in situ* lesions (pTis) to the advanced BCa stages T1–4 (Figure 1D). During the crucial step in BCa progression, the invasion into the detrusor muscle, MED1 protein expression was significantly downregulated compared to NMIBC (Figure 2A, *p* = 2.5E−07). Interestingly, low MED1 expression in the primary tumor was significantly associated with positive lymphonodal status (N1) (Figure 2B, *p* = 0.03) and showed a tendency toward the presence of distant metastases (M1) at the time of diagnosis (Figure 2C, *p* = 0.07). Patients with low MED1 protein expression showed an unfavorable clinical outcome and decreased survival evaluated by Kaplan–Meier estimator and log-rank test (Figure 2D, log-rank *p* = 0.02).

## Discussion

MED1 is part of the highly evolutionarily conserved multi-protein Mediator complex. Forming a bridge between transcriptional factors and the RNA Pol II, the Mediator complex is a central integrator of the transcriptional machinery. At the beginning of the transcriptional process it plays an important role in assembling the pre-initiation complex, which is formed on nucleosome-free templates. Further, through diverse functional interactions with chromatin cofactors the Mediator complex strongly contributes to chromatin remodeling (3–5). Interestingly, diverse subunits, especially MED1, were described to contribute to these gene-specific chromatin-remodeling processes (4, 20). The comprehensive molecular characterization of BCas by The Cancer Genome Atlas Research Network showed that chromatin regulatory genes were more frequently mutated in BCa than in any other common cancer, which suggests the chromatin structure to be highly relevant in BCa development (21). This indicates that the Mediator complex could serve a promising therapeutic target in BCa management.

The Mediator complex as an essential regulator of Pol II is localized in the nucleus. The immunohistochemical staining pattern for MED1 on our bladder tissue cohort showed MED1 to be preeminently nuclear but also in the cytoplasm, where protein-coding genes are translated (Figures 1A–C). The immunohistochemical staining pattern for MED1 on our bladder tissue cohort showed MED1 to be preeminently nuclear; therefore, we decided to assess the nuclear MED1 expression in our BCa cohort. Nevertheless, some subunits have additional functions besides their role in the Mediator complex and to evaluate a possible function for cytoplasmic or Mediator complex independent MED1 in the BCa biology further investigations are needed (22).

By performing immunohistochemical staining for MED1 on a large BCa cohort, we found significantly decreased MED1 expression during BCa development and progression to muscle-invasive and metastatic stages (Figures 1 and 2A–C). The presence of lymph node metastases was significantly associated with low MED1 expression in the primary tumor, whereas an association between MED1 expression and distant metastases remained non-significant probably due to low sample sizes (Figures 2B,C) (*p* = 0.07; pM1, *n* = 4). Further, low MED1 expression was associated with strongly reduced overall survival in our cohort (Figure 2D).

Differential expression of MED1, a subunit of the tail module, has also been frequently linked to other cancer types (10–17). Interestingly, in two independent studies, siRNA-mediated knockdown of MED1 in melanoma and non-small cell lung cancer cells led to enhanced invasive properties *in vitro* by modulating metastasis-related genes such as the urokinase receptor/uPAR (10, 12). In BCa, high levels of urokinase have been linked to enhanced motility of BCa cell lines and metastatic spread (23). Further, in lung adenocarcinoma, decreased MED1 expression was associated with diverse parameters of malignancy such as advanced pT stage, positive lymphonodal status, and shorter survival (11). Overall, downregulation of MED1 has been linked to enhanced invasion and tumorigenic phenotype in several cancer entities, which may support our observed association of MED1 with clinicopathological parameters of worse outcome in BCa (10–12) (Figure 2). In addition, the Mediator complex interacts directly with nuclear receptors such as the ER receptor and thereby enhances ER receptor function *in vitro* (14, 15). Interestingly, hyperactivity of MED1 has been described to influence tamoxifen resistance of human breast cancer cells in a HER2-dependent manner (16) and also has been suggested to promote prostate cancer oncogenesis (17). In total, MED1 seems to act in a tumor-specific manner, whereby MED1 up- or downregulation can enhance tumorigenicity. A possible explanation might be that MED1 as a coactivator of nuclear hormone receptors is necessary in hormone-dependent tumors like prostate or breast cancer. Whereas in other cancer entities such as melanoma, lung cancer as well as BCa downregulation of MED1 increases tumorigenic potential by modulating metastasis-related genes like uPAR.

## Conclusion

In conclusion, differential protein expression of the Mediator complex subunit MED1 was found in BCa specimen with significantly decreased expression in advanced metastatic muscle-invasive cancers. MED1 protein expression exhibited prognostic value as patients with low MED1 expression show shorter overall survival. In total, decreased MED1 expression may play a role during BCa progression, muscle invasion, and metastatic spread, but nevertheless functional investigations are needed to confirm this hypothesis.

## Authors Contributions

SP, NK, and IS designed the study approach and experiments. SP, NK, JB, and IS wrote the manuscript and performed microscopic and histopathologic investigations. NK, WV, JE, DS, and SM were responsible for clinical, microscopic, histopathologic elements and participated in pathological investigations. NK, IS, SP, JB, and DA were responsible for analysis and interpretation of the data. All authors read and approved the final manuscript.

## Conflict of Interest Statement

The authors declare that the research was conducted in the absence of any commercial or financial relationships that could be construed as a potential conflict of interest. The reviewers DN and HG and handling editor declared their shared affiliations, and the handling editor states that the process nevertheless met the standards of a fair and objective review.
